# The Natural Antimicrobial Carvacrol Inhibits *Campylobacter jejuni* Motility and Infection of Epithelial Cells

**DOI:** 10.1371/journal.pone.0045343

**Published:** 2012-09-25

**Authors:** Lieke B. van Alphen, Sara A. Burt, Andreas K. J. Veenendaal, Nancy M. C. Bleumink-Pluym, Jos P. M. van Putten

**Affiliations:** 1 Department of Infectious Diseases and Immunology, Utrecht University, Utrecht, The Netherlands; 2 Institute for Risk Assessment Sciences, Faculty of Veterinary Medicine, Utrecht University, Utrecht, The Netherlands; Institut National de la Recherche Agronomique, France

## Abstract

**Background:**

Natural compounds with anti-microbial properties are attractive reagents to reduce the use of conventional antibiotics. Carvacrol, the main constituent of oregano oil, inhibits the growth of a variety of bacterial foodborne pathogens. As concentrations of carvacrol may vary *in vivo* or when used in animal feed, we here investigated the effect of subinhibitory concentrations of the compound on major virulence traits of the principal bacterial foodborne pathogen *Campylobacter jejuni*.

**Methods/Principal Findings:**

Motility assays revealed that subinhibitory concentrations of carvacrol inhibited the motility of *C. jejuni* without affecting bacterial growth. Immunoblotting and electron microscopy showed that carvacrol-treated *C. jejuni* still expressed flagella. The loss of motility was not caused by reduced intracellular ATP levels. *In vitro* infection assays demonstrated that subinhibitory concentrations of carvacrol also abolished *C. jejuni* invasion of human epithelial cells. Bacterial uptake of invasive *Escherichia coli* was not blocked by carvacrol. Exposure of *C. jejuni* to carvacrol prior to infection also inhibited cellular infection, indicating that the inhibition of invasion was likely caused by an effect on the bacteria rather than inhibition of epithelial cell function.

**Conclusions/Significance:**

Bacterial motility and invasion of eukaryotic cells are considered key steps in *C. jejuni* infection. Our results indicate that subinhibitory concentrations of carvacrol effectively block these virulence traits by interfering with flagella function without disturbing intracellular ATP levels. These results broaden the spectrum of anti-microbial activity of carvacrol and support the potential of the compound for use in novel infection prevention strategies.

## Introduction

*Campylobacter jejuni* is the leading cause of bacterial foodborne disease and a major causative agent of traveler's diarrhea worldwide [1]–[3]. Symptoms of campylobacteriosis include diarrhea, fever, abdominal cramps and nausea. In a small group of patients serious sequela such as Guillain-Barré syndrome, an acute demyelinating disease of the peripheral nervous system [4], and reactive arthritis [5] contribute to the disease burden. Prolonged or severe *Campylobacter* infections and infected immunocompromised hosts need antibiotic therapy. However, increasing resistance to various antibiotics and concern for public health [6]–[8] strengthen the need for novel methods to reduce *Campylobacter* infections.

Main routes of acquiring *C. jejuni* infection are the consumption of contaminated meat, in particular poultry [9], and the ingestion of contaminated water [10]. Despite many advances in understanding *C. jejuni* infection, the exact mechanisms underlying intestinal colonization and disease are still poorly understood. At a cellular level, it is known that *C. jejuni* requires motility, mediated by its two polar flagella, for colonization and full virulence [11], [12]. Bacteria lacking flagella or expressing a non-motile flagellum show reduced colonization of epithelial cells *in vitro* [13], [14] and of the chicken intestinal tract *in vivo* [15]. Other factors implicated in virulence include several outer membrane proteins (including PEB1, JlpA, MOMP and CadF) [16]–[19], the phase variable capsule [20], [21], protein glycosylation [22] and lipooligosaccharide (LOS) [23]–[25] (for review: see [26]).

Novel strategies of fighting bacterial infections include the use of essential oil components with antibacterial activity. Carvacrol, a major constituent of oregano oil, is such a substance. It is an approved food flavor both in the US and in Europe and is currently also used as antibacterial feed additive and food preservative [27]. The bacterial target of carvacrol remains to be established. However, when added in bactericidal or growth-inhibitory concentrations, it can induce changes in the fatty acid composition [28], [29] and permeabilize the outer membrane of several Gram-negative species, resulting in leakage of ions and outer membrane-associated material to the medium [30]. Furthermore, in some species carvacrol possesses ATPase-inhibiting activity [31], [32] and has been proposed to act as a proton exchanger that reduces the pH gradient across the cytoplasmic membrane causing a collapse of the proton motive force and depletion of the ATP pool leading to cell death [33]. In *Bacillus cereus*, addition of carvacrol below the minimum inhibitory concentration (MIC) sharply reduces toxin production, suggesting that lower concentrations of carvacrol may contribute to food safety as well [34].

Most studies on the effects of carvacrol on bacteria have focused on determining the MIC at which growth arrest of the bacterial culture occurs, or on the minimum bactericidal concentration (MBC) i.e. the concentration at which >99.9% of the bacterial population is killed. In the present study, we investigated the potential effects of sub-inhibitory concentrations (subMIC) of carvacrol on the virulence potential of *C. jejuni*. Exposure of pathogens to subinhibitory concentrations of the compound are likely to occur when added to animal feed or when used during decontamination e.g. during slaughter. Our results indicate that at concentrations that do not affect bacterial growth carvacrol inhibits bacterial motility and invasion of eukaryotic cells which may limit bacterial spread and infection.

## Materials and Methods

### Bacteria and cell culture

*C. jejuni* strains 108 (129108, 108WT) [35] and 81116 [36] were routinely grown on saponin agar plates with 6% lysed horse blood (Biotrading) or in 5 ml of Heart Infusion (HI) broth (Biotrading) under microaerophilic conditions at 37°C. *E. coli inv*, expressing the invasin of *Y. pseudotuberculosis* [37], was routinely grown on LB agar plates at 37°C. INT-407 (CCL-6, ATCC) intestinal epithelial cells were routinely maintained in Dulbecco's minimal essential medium (DMEM) supplemented with 10% fetal bovine serum (FBS) in a humidified 37^o^C atmosphere of 10% CO_2_.

### Growth curves

Carvacrol (Sigma) was dissolved in 96% ethanol to a final concentration of 0.1 M prior to use. The subMIC for *C. jejuni* (highest concentration of carvacrol that does not inhibit bacterial growth) was determined by measuring the optical density (550 nm) of cultures (final start OD_550_: 0.05) after incubation under microaerophilic conditions (16 h, 160 rpm, 37°C). To quantify the number of bacteria, serial dilutions were plated, incubated (24 h), and colonies enumerated. Growth kinetics of *C. jejuni* were determined in HI broth in the absence and presence of 0.2 mM carvacrol by measuring OD_550_ of the culture at indicated intervals.

### Construction of motility deficient mutants

The *C. jejuni motAB* mutant was constructed as described [38]. PCR was used to amplify the *motAB* genes and their flanking regions using primers 335fw (5′-TATGATGAGTTTTATGGGCGAGAG-3′) and 338rev (5′-TTGATACCGAAACCACAGGACTTG-3′). This PCR fragment was cloned into the pGEM-Teasy vector in *E. coli* DH5α. Reverse PCR on the pGEM*motAB*, using outward oriented primers motB-Bgl-out (5′-CCCAGATCTTAGGAACTAAAGCACCTTAAAAG-3′) and motA-Bgl-out (5′-CCCAGATCTGTTGAAAGATCCATTAATTATCTCC-3′) was used to delete 1489 bp from *motAB* and to introduce a unique *Bgl*II restriction site that served to insert the Km^r^ cassette from pMW2, yielding pGEM*motAB*:km. This knockout construct was transformed into *C. jejuni* 81116 and *C. jejuni* 108 to generate *ΔmotAB*. Double cross-over recombination events were confirmed by PCR.

### Electron microscopy

A drop of bacteria (strain 108) grown in HI broth (16 h) in the presence or absence of 0.2 mM of carvacrol was added onto a 100-mesh grid, coated with 0.7% FormVar (Agar Scientific, Essex, UK). After 10 min, the grids were stained with 2% tungstophosphoric acid for 1 s and allowed to dry. The grid was viewed in a Philips CM10 electron microscope.

### Immunoblotting

To assess protein profiles and flagellin expression, *C. jejuni* strain 108 was grown overnight in HI broth and then diluted to an optical density at 550 nm of 0.10 into 5 ml of HI broth containing either solvent (ethanol) or various concentrations of carvacrol. After 2 h of incubation (37°C, microarobic conditions, 160 rpm), equal amounts of bacteria were collected (2,000×*g*, 5 min), mixed with Laemmli buffer, and heated (95°C, 10 min). Aliquots were separated by SDS-PAGE and either stained with Coomassie Brilliant Blue or immunoblotted onto PVDF membranes. Blots were incubated with *C. jejuni* flagellin rabbit antiserum 313 and, subsequently, with goat-anti rabbit IgG-peroxidase. Blots were developed with Supersignal West Pico chemiluminescent substrate (Pierce Biotechnology).

### Motility assays and time-lapse microscopy

To assess bacterial motility in the presence or absence of sub-inhibitory concentrations of carvacrol the well-established soft-agar method was used [39]. In brief, equal numbers of bacteria of *C. jejuni* strains 108, 81116, 108Δ*motAB* or 81116 Δ*motAB* grown in HI broth were pipetted into semi-solid medium (thioglycollate medium with 0.4% agar) containing up to 0.1 up to 0.4 mM of carvacrol or an equivalent amount of solvent (ethanol). Bacterial migration through the semi-solid agar was assessed after incubation (24 h) under microaerophilic conditions at 37°C, as described [39].

For dark-field and time-lapse microscopy, *C. jejuni* strain 108 grown in HI broth in the absence or presence of various concentrations of carvacrol was observed at 1000x magnification under a phase contrast microscope for 1 min and classified by a method established by Gill and Holley [40]. Suspensions which were actively moving and tumbling were classed as motile; suspensions in which a minority of cells of cells were either moving or tumbling were classed as having reduced motility; and suspensions showing only stationary vibrational movement were classed as non motile. In addition, the presence of the coccoid form was noted. Motility was recorded using an Olympus DP25 camera.

### ATP measurements

To determine intracellular ATP levels, HI grown (16 h) *C. jejuni* strain 108 was collected by centrifugation (3,000×g, 10 min, 20°C) and resuspended in HEPES buffered saline (10 mM HEPES, 5.4 mM KCl, 145 mM NaCl, 1 mM MgCl_2_, 1 mM CaCl_2_, pH 7.4) to OD_550_ of 0.05. When appropriate, 20 mM of L-serine, 0.2 mM of carvacrol (or the equivalent amount of solvent ethanol) or 5 μM of carbonyl cyanide-m-chlorophenylhydra­zone (CCCP) were added as supplements. After 2 h of incubation at 37°C under microaerophilic conditions, 1 ml of suspension was centrifuged (3,000×g, 5 min, 20°C) and the cell pellet resuspended in 100 μl of lysis buffer (100 mM Tris, 4 mM EDTA, pH 7.75). Subsequently, 900 μl of boiling lysis buffer was added. Samples incubated for 2 min at 100°C, placed on ice, and centrifuged (1,000×g, 1 min, 4°C). One-hundred microliters of supernatant was mixed with an equal amount of luciferase reagent from the ATP Bioluminescence Assay CLS II kit (Roche) and luciferase activity was measured using a luminometer (TD-20/20, Turner Designs). The ATP concentration was calculated from an ATP standard curve.

### Infection and gentamicin protection assay

Infection experiments were carried out as described [41] with some modifications. Briefly, epithelial cells (75–80% confluence) were rinsed twice with serum-free medium and placed in 1 ml of this medium in a microaerobic incubator at 30 min prior to infection. Bacteria, grown in HI broth for 16 h (OD_550_: 1.2) in the presence or absence of 0.2 mM of carvacrol, were collected by centrifugation (6,000×*g*, 5 min, 20°C), resuspended in Dulbecco's phosphate buffered saline (DPBS), and added to the cells at an m.o.i. of 200. Where indicated, the plates were centrifuged (600×*g*, 10 min, 20°C). Bacterial invasion was assessed after 2 h of infection using the gentamicin protection assay. Cells were first rinsed three times with 1 ml of DPBS, then incubated (3 h, 37°C) in 0.5 ml of medium containing 250 μg/ml of gentamicin, rinsed three times with 1 ml of DPBS, lysed with 250 μl of 0.1% Triton X-100 in DPBS (15 min, 20°C), and plated at various dilutions to quantify the number of viable bacteria. Triton X-100 did not influence *C. jejuni* recovery irrespective of carvacrol treatment. No viable bacteria were recovered from the culture supernatant after gentamicin treatment, indicating that the treatment was effective. Experiments were performed in duplicate and the averages (mean ± SEM) of three separate experiments are presented.

### Statistical analysis

Results were analyzed for statistical differences by Student's unpaired *t*-test, one-way or two-way ANOVA where appropriate. Statistical significance was accepted at *p*<0.05.

## Results

### Effect of carvacrol on *C. jejuni* growth

**Figure 1:**
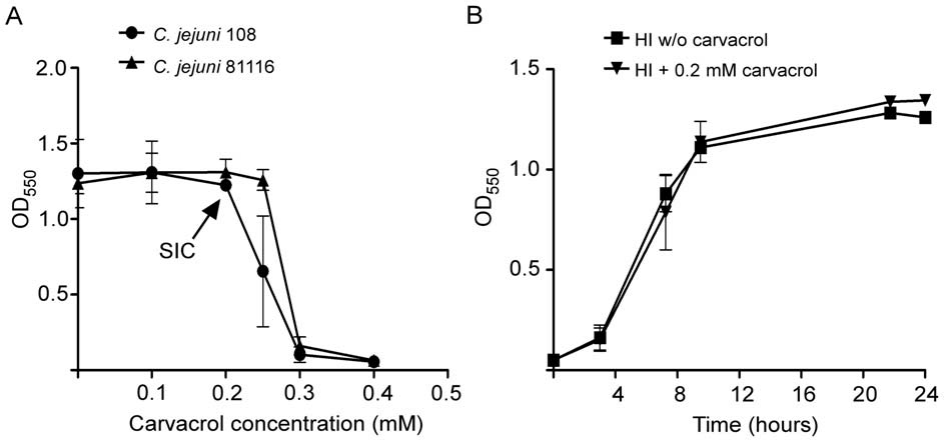
Effect of carvacrol on *C. jejuni* growth. (A) Growth of *C. jejuni* strains 108 and 81116 in HI supplemented with increasing concentrations of carvacrol as determined after 16 h of incubation. The maximum sub-inhibitory concentration (SIC) is indicated by an arrow. (B) Growth kinetics of *C. jejuni* strain 108 with or without carvacrol at SIC monitored during a 24 h-period. Results are mean values ± standard error of three independent experiments.

Most studies on the effects of carvacrol on *C. jejuni* have focused on the concentrations necessary to partially (MIC) [42] or totally (MBC) [43] inhibit bacterial growth. To investigate whether sub-inhibitory concentrations (subMIC) of carvacrol influence virulence traits of *C. jejuni*, we first determined the highest concentration of carvacrol that did not inhibit growth of *C. jejuni* strains 108 and 81116. Bacterial growth was determined after 16 h of incubation under microaerobic conditions in the presence of various concentrations of carvacrol. The highest sub-inhibitory concentration of carvacrol for strains 108 and 81116 was 0.2 mM and 0.25 mM respectively, both when bacteria were grown in HI broth (Fig. 1A) and in tissue culture medium consisting of DMEM enriched with 5% FCS (data not shown). At higher concentrations (≥0.4 mM), bacterial growth was inhibited as judged from the reduced OD_550_ levels reached after 16 h of incubation (Fig. 1A). Time course experiments performed with strain 108 showed similar growth kinetics in the absence and presence of 0.2 mM of carvacrol during the entire period of incubation (Fig. 1B). Enumeration of colony forming units confirmed the presence of equal numbers of bacteria for both cultures (data not shown).

### Effect of carvacrol on flagella formation and motility

**Figure 2:**
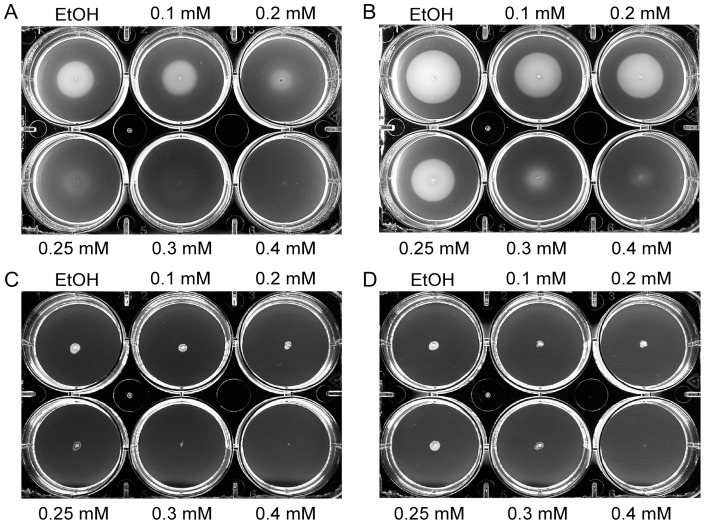
Inhibition of *C. jejuni* motility by carvacrol. Swarming of *C. jejuni* strain 108 (A) and 81116 (B) and the non-motile mutants 108Δ*motAB* (C) and in 81116Δ*motAB* (D) in thioglycollate agar in the absence and presence of the indicated concentrations of carvacrol after 24 h of growth. The carvacrol solvent ethanol (EtOH) served as negative control.

**Figure 3:**
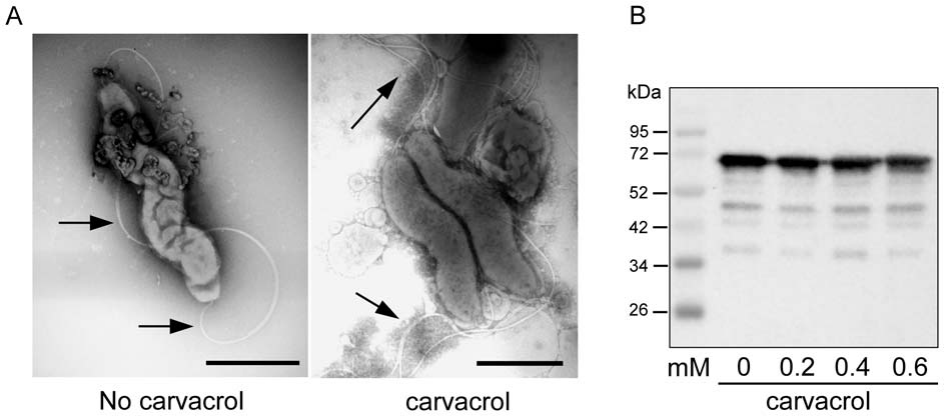
Effect of carvacrol on flagella formation. (A) Representative electronmicrographs of flagella-expression by *C. jejuni* strain 108 (arrows) grown in the absence (left panel) and presence (right panel) of 0.2 mM of carvacrol. Bar represents 1 µm. (B) Western blot of whole cell lysates of *C. jejuni* strain 108 grown at the indicated concentrations of carvacrol. The blot was probed with rabbit anti-flagella antiserum 313 and subsequently with goat-and-rabbit IgG-peroxidase and substrate. Molecular mass markers are indicated in kilodaltons (kDa).

Flagella-driven bacterial motility is an important virulence trait of *C. jejuni*. The effect of sub-inhibitory concentration of carvacrol on *C. jejuni* motility was first assessed for different concentrations of carvacrol. *C. jejuni* using dark-field microscopy (Table 1). Time-lapse microscopy confirmed that *C. jejuni* strain 108 became non-motile when grown in HI broth in the presence of 0.2 mM of carvacrol (see Supporting Information, Movie S1 and S2). Strain 81116 became non-motile at 0.25 mM of carvacrol. At concentrations >0.4 mM of carvacrol, both strains gained a coccoid appearance (Table 1). Similar observations were made for bacteria kept in DMEM with carvacrol. The lack of motility was verified in a motility assay in 0.4% thioglycollate soft-agar plates containing increasing concentrations of carvacrol. Again, carvacrol inhibited bacterial migration, while strong migration of the strains was observed in the absence of carvacrol (Fig. 2A and B). The inhibition of the movement in the semi-solid medium required slightly higher concentrations of carvacrol than in HI broth most likely due to the limited diffusion of the compound in the soft-agar. Electron microscopy on *C. jejuni* grown in HI broth in the absence or presence of 0.2 mM of carvacrol showed that the bacteria carried polar flagella that appeared morphologically similar irrespective the presence of carvacrol (Fig. 3A). Thus, carvacrol seemed to inhibit flagella function rather than flagella biosynthesis. Immunoblotting confirmed the presence of similar amounts of bacterial flagellin in the absence and presence of 0.2 mM of carvacrol (Fig. 3B).

**Table 1:** Effect of carvacrol on the motility and physiology of *C. jejuni* strains 108 and 81116 cultured (16 h) in HI broth with shaking in the absence or presence of the indicated concentrations of carvacrol.

Carvacrol (mM)	*C. jejuni* 108	*C. jejuni* 81116
0	motile	motile
0.1	motile	motile
0.2	non motile	motile
0.25	non motile, some coccoid	non motile
0.3	non motile, some coccoid	non motile
0.4	only coccoid	only coccoid

Motility and morphology were scored by dark-field microscopy.

### Effect of carvacrol on *C. jejuni* ATP levels

**Figure 4:**
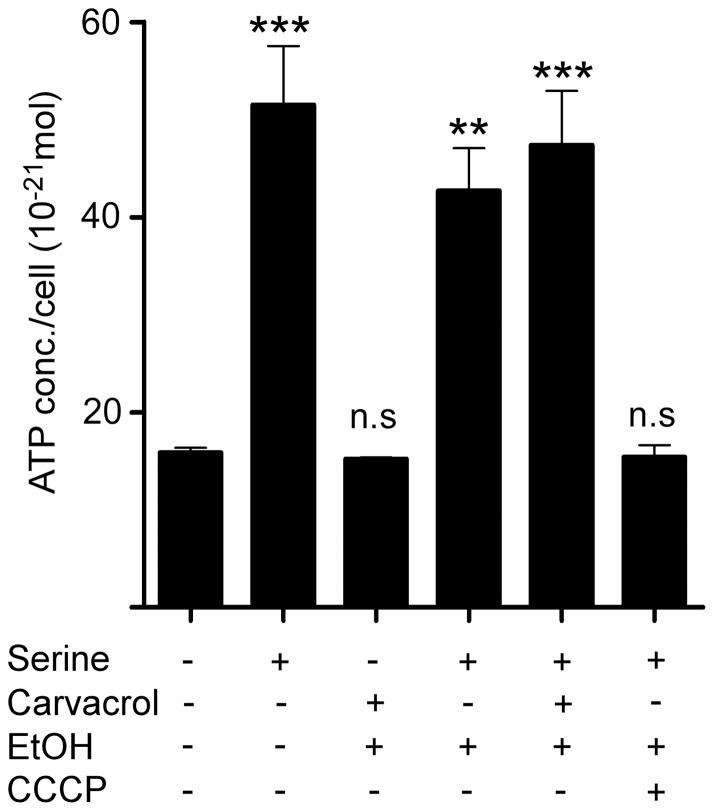
Effect of carvacrol on *C. jejuni* ATP levels. *C. jejuni* strain 108 grown in HI broth (16 h) was resuspended in HEPES buffer with or without the indicated supplements. After 2 h incubation at 37°C under microaerophilic conditions, bacterial ATP levels were determined. Results are expressed as the ATP concentration per bacterium and are mean values ± standard error from three independent experiments. ** *p*≤0.005; *** *p*≤0.001; n.s. not statistically significant.

Previously, high concentrations of carvacrol have been shown to deplete intracellular ATP levels in *B. cereus*, resulting in cell death [44]. To determine whether the impaired *C. jejuni* motility was caused by depletion of ATP, bacterial ATP levels were measured using a luciferase assay. This assay demonstrated profoundly reduced ATP levels for *C. jejuni* strain 108 kept in Hepes buffer compared to Hepes buffer with L-serine as a carbon source (Fig. 4). In the presence of L-serine and carvacrol (0.2 mM), similar ATP levels were detected as with L-serine alone, indicating that carvacrol does not reduce ATP levels in *C. jejuni* (Fig. 4). Even at growth-inhibitory concentrations of carvacrol (0.4 mM), no reduction of *C. jejuni* ATP levels was observed (data not shown). A reduction in ATP levels was detected after addition of carbonyl cyanide-m-chlorophenylhydrazone (CCCP), a disruptor of the proton motive force (Fig. 4). This effect was already observed at a concentration (5 μM) that did not inhibit *C. jejuni* growth. Thus, carvacrol inhibits *C. jejuni* motility independent of bacterial ATP levels.

### Effect of carvacrol on invasion of INT-407 cells by *C. jejuni*

**Figure 5:**
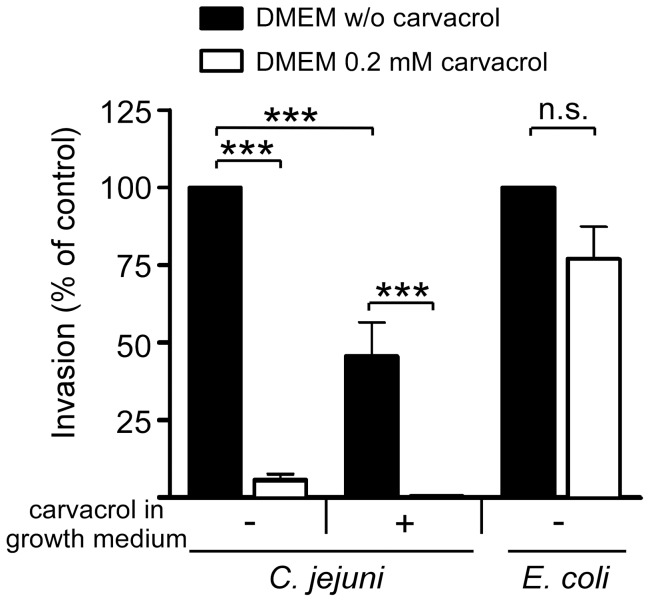
Effect of carvacrol on bacterial invasion of INT-407 cells. Invasion of INT-407 cells by *C. jejuni* (strain 108) and *E. coli inv* in the absence (filled bars) and presence (open bars) of 0.2 mM of carvacrol. Bacteria used for inoculation were grown in HI broth without (−) or with (+) carvacrol. Invasion was determined using the gentamicin protection assay. Note that the reduction in invasion was specific for *C. jejuni* and most effective when carvacrol was present before and during the infection assay. Results are expressed as percentage of invasion in the absence of carvacrol and are mean values ± standard error from at least three independent experiments. *** *p*≤0.001; n.s. not statistically significant.

As flagella are important for *C. jejuni* invasion of eukaryotic cells and intestinal colonization [12], we next investigated the effect of carvacrol on the invasion properties of *C. jejuni*. Bacterial entry into cultured epithelial INT-407 cells was measured using a standard gentamicin protection assay [41]. Addition of carvacrol (0.2 mM) during infection (i.e. not during the *C. jejuni* preculture) reduced *C. jejuni* invasion levels to as low as 5.6%±1.9% of the invasion observed in the absence of the compound (Fig. 5). When carvacrol was added both during *C. jejuni* growth and subsequent infection of INT-407 cells, invasion levels further reduced to approximately 0.38%±0.09% (Fig. 5).To determine whether the presence of carvacrol during bacterial growth only was already sufficient to induce a *C. jejuni* invasion-deficient phenotype, bacteria were grown (16 h) with 0.2 mM of carvacrol, washed, and incubated with INT-407 cells in the absence of carvacrol. This procedure caused a 45.6%±10.8% reduction of bacterial invasion compared to *C. jejuni* grown in the absence of carvacrol (Fig. 5).

To further establish that carvacrol acted on the pathogen rather than on the uptake capacity of the INT-407 cells, we determined the effect of carvacrol on the invasion of recombinant *E. coli* expressing the invasin of *Yersina pseudotuberculosis* [37]. This strain efficiently enters epithelial cells via β1-integrin receptors [45]. Carvacrol caused a minimal decrease in invasion of *E. coli inv* compared to the drastic reduction of *C. jejuni* invasion (Fig. 5). This result indicates that carvacrol does not cause a general defect in bacterial uptake in the eukaryotic cells, but specifically inhibits *C. jejuni* infection.

### Partial restoration of invasion in carvacrol-treated *C. jejuni*

**Figure 6:**
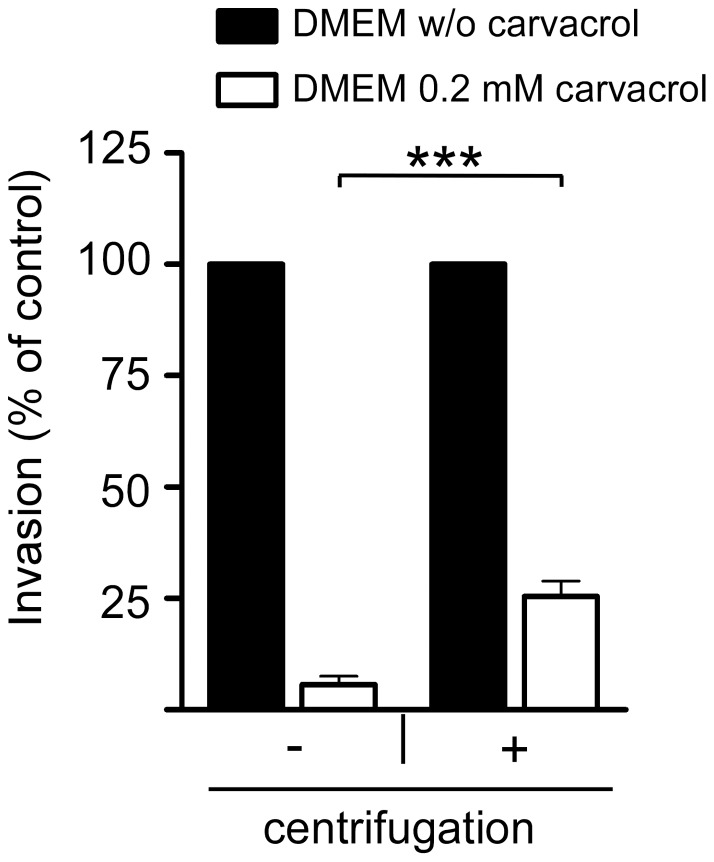
Partial restoration of invasion in carvacrol-treated *C. jejuni*. Invasion of *C. jejuni* (strain 108) into INT-407 cells was determined in the absence (filled bars) and presence (open bars) of 0.2 mM of carvacrol, without (−) and with (+) centrifugation of the bacteria onto the cells at the start of the infection. Note the five-fold increase in invasion by *C. jejuni* grown with carvacrol after centrifugation. Results are expressed as percentage of invasion in the absence of carvacrol and are mean values ± standard error of at least three independent experiments. *** *p*≤0.001.

To substantiate the lack of bacterial motility as the cause of the inhibition of bacterial invasion, we performed infection assays in which the bacteria were centrifuged onto the cells, which reduced the requirement for motility to reach the cell surface. Centrifugation of the bacteria onto the cells restored the *C. jejuni* invasion in the presence of carvacrol from 5.6% (±1.9%) to 25.5% (±3.5%) of the invasion levels observed without carvacrol (Fig. 6). This finding indicates that the inhibition of *C. jejuni* invasion by carvacrol is at least partially caused by the reduction in bacterial motility.

## Discussion

Natural compounds that limit the spread and virulence of bacterial foodborne pathogens may be attractive alternatives for conventional antimicrobials. For their application as disinfectants and in therapy, it is important to establish the concentration range and mechanism of the anti-microbial activity. Carvacrol is a major component of oregano oil that inhibits the growth of many food-borne pathogens including emerging antibiotic-resistant *C. jejuni* [42], [43]. Carvacrol has been approved as food flavor in both the USA and Europe, is used as antibacterial feed additive and food preservative [27], and may be used as decontaminant e.g. during slaughter. During these applications local drug concentrations may show large variations which may influence the efficacy of the treatment.

Here we investigated the effect of sublethal and sub-inhibitory concentrations of carvacrol on bacterial behavior to assess its spectrum of biological activities. Our results indicate that even at concentrations that do not impair bacterial growth, carvacrol attenuates *C. jejuni* virulence and protects against cellular infection. Titration of the effect of carvacrol on bacterial growth revealed that a concentration of ≥0.4 mM was sufficient for complete growth inhibition of *C. jejuni* strains 108 and 81116. Sub-inhibitory concentrations (here defined as the highest concentration that did not reduce bacterial growth) were determined at 0.2 mM of carvacrol for *C. jejuni* 108 and 0.25 mM for *C. jejuni* 81116 (Fig. 1A). At these concentrations, no difference in growth kinetics could be observed between cultures with or without carvacrol (Fig. 1B), but bacterial motility and bacterial invasion of eukaryotic cells were virtually abolished.

Electron microscopy showed *C. jejuni* with flagella after carvacrol treatment. These results in combination with the measured unaltered flagellin expression as determined by immunoblotting (Fig. 3B) indicate defective flagella function rather than a lack of filament assembly as the most likely molecular basis of the motility defect. This result was unexpected as for *E. coli* O157:H5 sublethal concentrations of carvacrol have been reported to reduce flagellar synthesis [46]. Due to its hydrophobic properties, carvacrol is predicted to influence the function of biological membranes. It is generally assumed that carvacrol can enter into the cytoplasm of bacterial cells and, at higher concentrations, affects the membrane integrity of *E. coli* and *Listeria monocytogenes* [47]. For *Bacillus cereus*, the bactericidal effect of carvacrol has been attributed to a depletion of intracellular ATP and a dissipation of ion gradients due to increased permeability of the cytoplasmic membrane [33], [44]. In our hands, carvacrol (0.2–0.4 M) did not reduce internal ATP levels of *C. jejuni*, although such an effect was measured after treatment with a sub-inhibitory concentration of the proton motive force disrupting compound carbonyl cyanide m-chlorophenylhydrazone (CCCP) (Fig. 4). These results indicate that carvacrol inhibits flagellar motility of *C. jejuni* without depleting bacterial ATP levels.

Gentamicin-protection assays revealed that 0.2 mM of carvacrol was sufficient to inhibit invasion of INT-407 cells by *C. jejuni* strain 108. The effect of carvacrol was most effective when the compound was present during the infection assay. Carvacrol is known to activate the Ca^2+^-permeable cation channel TRPV3 that acts as a receptor in oral and nasal epithelium [48]. The reduction in *C. jejuni* invasion may thus have been caused by an effect on epithelial cell behavior. However, we consider an effect of carvacrol on these eukaryotic uptake pathway(s) unlikely as (partial) inhibition of invasion was also observed when *C. jejuni* was pre-treated with carvacrol. Additionally, the non-motile but highly invasive *E. coli inv* [39] was still taken up efficiently by the cells in the presence of carvacrol, suggesting that at least the uptake pathway used by this bacterium is intact in the presence of carvacrol. This scenario is in line with studies that report the bactericidal activity of carvacrol to occur at far lower concentrations than cytotoxic effects on mammalian cells [49]. During the time span of the infection assay, we observed no effects of carvacrol on cellular apoptosis and proliferation. Furthermore, the concentrations of carvacrol used in this study approximate the lowest concentration used in some animal feed studies [50], although 10- to 50-fold higher concentrations are used more commonly [51]. The absence of apparent cytotoxicity and the observed partial restoration of *C. jejuni* invasion in the presence of carvacrol when the bacteria were centrifuged onto the cells further suggest that the lack of bacterial motility largely accounts for the reduction in invasion. Functional flagella are well known to be required for efficient entry of *C. jejuni* into eukaryotic cells [13]–[15], [39].

In conclusion, the present results show for the first time that sub-inhibitory concentrations of carvacrol inhibit *C. jejuni* motility and invasion into eukaryotic cells. As flagella-mediated motility is required for *C. jejuni* virulence and the colonization of poultry, our results support and may widen the window for the use of carvacrol as an antibacterial additive or as supplement in animal feed.

## Supporting Information

Movie S1 **Time-lapse microscopy showing the movement of** ***C. jejuni*** **strain 108 in the absence of 0.2 mM of carvacrol.**
(WMV)

Movie S2 **Time-lapse microscopy showing the movement of** ***C. jejuni*** **strain 108 in the presence of 0.2 mM of carvacrol.**
(WMV)
